# Upregulation of miR-29b-3p protects cardiomyocytes from hypoxia-induced apoptosis by targeting TRAF5

**DOI:** 10.1186/s11658-019-0151-3

**Published:** 2019-04-11

**Authors:** Yuhua Cai, Yunpeng Li

**Affiliations:** 1 Department of Cardiovasology, Jingzhou First Municipal Hospital, Jingzhou, Hubei Province China; 2 Department of Cardiovasology, Dongfeng Hospital, Hubei University of Medicine, No. 16 Daling Road, Shiyan, 442008 Hubei Province China

**Keywords:** Cardiomyocyte, Hypoxia, miR-29b-3p, TRAF5

## Abstract

**Background:**

MicroRNAs (miRNAs) are pivotal regulators in regulating hypoxia-induced cardiomyocyte injury. This study was designed to evaluate the effects of miR-29b-3p on hypoxic cardiomyocytes.

**Methods:**

Human AC16 cells were cultured under normoxic or hypoxic conditions. Hypoxic injury was confirmed based on alterations in cell viability using CCK-8 assay and apoptosis using flow cytometry and Hoechst staining. Bioinformatics analyses and the dual-luciferase reporter assay were performed to predict and validate the target gene of miR-29b-3p.

**Results:**

We found that hypoxia suppressed cell viability and promoted apoptosis. TNF receptor-associated factor 5 (TRAF5) was a potential target gene of miR-29b-3p. Our in vitro experiments revealed that miR-29b-3p overexpression or TRAF6 knockdown significantly protected cardiomyocytes against hypoxia-induced injury. Moreover, knockdown of TRAF5 knockdown potentiated the protective effects of miR-29b-3p against hypoxia-induced cell injury.

**Conclusion:**

These findings suggest that upregulation of miR-29b-3p could protect cardiomyocytes against hypoxia-induced injury through downregulation of TRAF5. Targeting TRAF5 with miR-29b-3p might be a potential therapeutic method for AMI.

## Background

Acute myocardial infarction (AMI) remains a major cause of death in both developed and developing countries [1]. A number of studies have pointed that AMI is associated with insufficient myocardial oxygen supply and increased myocardial workload [2]. When AMI occurs, the heart undergoes molecular, structural, genomic and functional changes as well as ventricular remodeling [3]. Despite some progress having been made with invasive strategy, primary percutaneous coronary intervention and antiplatelet agents, there is still a high incidence of heart failure in AMI patients [4]. It is widely accepted that ischemic hypoxia-induced cardiomyocyte apoptosis is the main reason for AMI [5]. Therefore, focusing on the molecular mechanisms involved in hypoxia-induced apoptosis may reveal a new treatment possibility.

MicroRNA (miRNA) is a type of small non-coding RNA between 20 and 23 nucleotides (nt) long. They function as post-transcriptional modulators of gene expression by accelerating messenger RNA degradation and repressing protein synthesis in a sequence-specific manner [6].

Some miRNAs have been implicated in the pathogenesis of AMI [7]. Recent studies revealed that miRNA dysregulation caused by genetic or epigenetic defects could induce or suppress cardiomyocyte cell apoptosis [8]. MiRNAs regulate the regeneration of cardiac muscle, control cardiomyocyte proliferation and alter stem or progenitor cell-mediated cardioprotective effects [9]. Numerous miRNAs, including miR-1 [10], miR-21 [11], miR-146a [11], miR-214 [12], and miR-208 [13], have been reported to be important factors in AMI.

MiR-29b-3p was recently shown to play key roles in malignancies [14], osteoarthritis [15] and cardiac fibrosis formation [16]. A mouse chronic kidney disease model revealed that upregulation of miR-29b-3p contributes to a reduction in the cardiac fibrosis caused when pNaKtide antagonizes Na/K-ATPase signaling [16]. However, the functional role of miR-29b-3p in AMI remains elusive.

Tumor necrosis factor (TNF) receptor-associated factors (TRAFs) are a family of critical adaptors that participate in many biological processes, such as inflammation, apoptosis, cell proliferation, autophagy and cytokine synthesis [17, 18]. TRAFs were identified as signal transducers of the TNF receptor (TNFR), TLR, IL-1 receptor and RIG-I-like receptor families [19].

TRAF5 contains an N-terminus RING finger domain [20] and is involved in TNF-induced NF-kB activation [21]. A recent study reported that TRAF5 is targeted by miR-26b and participates in melanoma cell proliferation and apoptosis by regulating MAPK activation [22]. The role of TRAF5 in AMI has rarely been investigated, but as it has pro-apoptotic signaling roles, we hypothesized that it may play an important role in cardiomyocyte apoptosis.

Here, we studied the expression of miR-29b-3p in hypoxic human AC16 cardiomyocyte cells. The function and the molecular mechanism of the miR-29b-3p–TRAF5 axis implicated in cardiomyocyte apoptosis were also investigated.

## Materials and methods

### Cell culture and treatments

Cells of the human AC16 cardiomyocyte line and 293 T embryonic kidney line were purchased from American Type Culture Collection (ATCC). The AC16 cells were cultured in Dulbecco’s modified Eagle medium (DMEM) with nutrient mixture F-12 (Sigma-Aldrich) and 10% FBS (Gibco). The 293 T cells were grown in DMEM with 10% FBS (Gibco). For the normoxia group, the cell lines were maintained in a normoxic incubator (21% O_2_, 5% CO_2_ and 74% N_2_) at 37 °C as normoxia group. For the hypoxia group, AC16 cells were incubated for 24 h in a hypoxic incubator (1% O_2_, 5% CO_2_ and 94% N_2_). This imitated myocardial ischemia.

### Oligonucleotide transfection

Hsa-miR-29b-3p mimics (miR-29b-3p), negative control oligonucleotide (miR-NC), small interfering RNA targeting TRAF5 (siTRAF5) and siNC were synthesized by GenePharma. For gain-of-function and loss-of-function experiments, AC16 cells were seeded in 6-well plates for 24 h and transfected with miR-29b-3p, miR-NC, siTRAF5 or siNC before being cultured under hypoxic conditions using Lipofectamine 2000 reagent (Invitrogen) according to the manufacturer’s instructions.

### RNA isolation and quantitative real-time PCR assay

Total RNA was isolated from cells using TRIzol reagent (Invitrogen). For quantitative real-time PCR, cDNA was synthesized from RNA using a TaqMan MicroRNA Reverse Transcription kit (Applied Biosystems) or M-MLV Reverse Transcriptase (BioTeke). The expressions of miR-29b-3p or TRAF5 were determined with SYBR Green (Takara) on a 7900HT Fast Real-Time PCR system (Applied Biosystems) using the primer sequences: miR-29b-3p forward: 5′-TGCGGTAGCACCATTTGAAAT-3′ and reverse: 5′-CCAGTGCAGGGTCCGAGGT-3′; and TRAF5 forward: 5′-CCGAGCCCCACAATGGCTTA-3′ and reverse: 5′-CCGCTCCACAAACTGGTACT-3′. The PCR conditions were: an initial denaturation at 95 °C for 7 min; 40 cycles of denaturation at 95 °C for 15 s, annealing at 60 °C for 30 s, and extension at 72 °C for 30 s. The relative expressions of the genes were calculated using the comparative delta-delta CT (2^-∆∆Ct^) method. Results were normalized to the expression of U6 (for miR-29b-3p) or i-actin (for TRAF5).

### Cell viability assay

Cell viability was assessed using a Cell Counting Kit-8 (CCK-8, Dojindo Molecular Technologies). Briefly, AC16 cells were seeded in 96-well plates at a density of 5000 cells per well. At 0, 24, 48, 72 and 96 h, 10 μl of CCK-8 reagent was added to the culture medium and the cultures were incubated for another 2 h at 37 °C. Finally, the optical density value at 450 nm was detected with a microplate reader (Bio-Rad).

### Flow cytometry analysis

Cell apoptosis analysis was performed using flow cytometry with an Annexin V/PI apoptotic detection kit (Beyotime Institute of Biotechnology). Briefly, differently treated AC16 cells were washed in phosphate-buffered saline (PBS) and re-suspended in binding buffer containing 10 μl of Annexin V and 5 μl PI according to the manufacturer’s instructions. The apoptotic cells were analyzed using a flow cytometry (BD Biosciences) equipped with FlowJo software (Tree Star).

### Hoechst staining

Hoechst staining was used to detect apoptosis of AC16 cells after the different treatments. Briefly, AC16 cells were seeded into 6-well plates and incubated overnight at 37 °C. After fixing with 50 μl cold 4% formaldehyde for 30 min, the cells were washed twice with cold PBS and incubated with 20 μg/ml Hoechst 33258 (Sigma-Aldrich) for 20 min at room temperature. Subsequently, the morphological changes of apoptosis were observed and identified as strong blue fluorescence under a Leica confocal laser-scanning microscope (Leica Microsystems).

### Luciferase reporter assay

The fragment from the human TRAF5 3′-untranslated region (3’UTR) containing the predicted miR-29b-3p-binding site was amplified via PCR and then cloned into pmirGLO vector (Promega) to form the reporter vector TRAF5-wild-type (WT TRAF5). To mutate the putative binding site of miR-29b-3p in the TRAF5 3’UTR, site-mutations were performed to generate TRAF5-mutated-type (MUT TRAF5). Then 293 T cells were transfected with the vectors WT TRAF5 or MUT TRAF5 together with miR-29b-3p or miR-NC using Lipofectamine 2000 (Invitrogen). After transfection for 48 h, the luciferase activity was measured using the Dual Luciferase Reporter Assay System (Promega).

### Western blotting

Total proteins were extracted from cells using RIPA lysis buffer (Beyotime Biotechnology) and quantified using the BCA Protein Assay Kit (Pierce) according to the manufacturer’s protocol. After quantification, 30 μg of total protein was separated via sodium dodecyl sulfate polyacrylamide gel electrophoresis (SDS-PAGE) and then transferred onto polyvinylidene fluoride (PVDF) membranes (Millipore). The membrane was blocked with 5% non-fat dry milk and then probed with antibodies against TRAF5 (1:1000, ab137763, Acbam) and GAPDH (1:5000, ab9482, Abcam) at 4 °C overnight, followed by incubation with horseradish peroxidase-conjugated secondary antibodies (Cell Signaling Technology). The protein bands were visualized using an ECL western blotting kit (Pierce).

### Statistical analysis

All experiments were performed in triplicate and repeated at least three times. The statistical analysis was carried out using SPSS package version 18.0 software (SPSS Inc.) and the data are expressed as means ± standard deviation (SD). Differences between two groups were evaluated using Student’s t-test and among multiple groups using one-way analysis of variance followed by Tukey’s test. *p* < 0.05 was considered statistically significant.

## Results

### Hypoxia induces cell injury and downregulates miR-29b-3p in AC16 cells

**Fig. 1:**
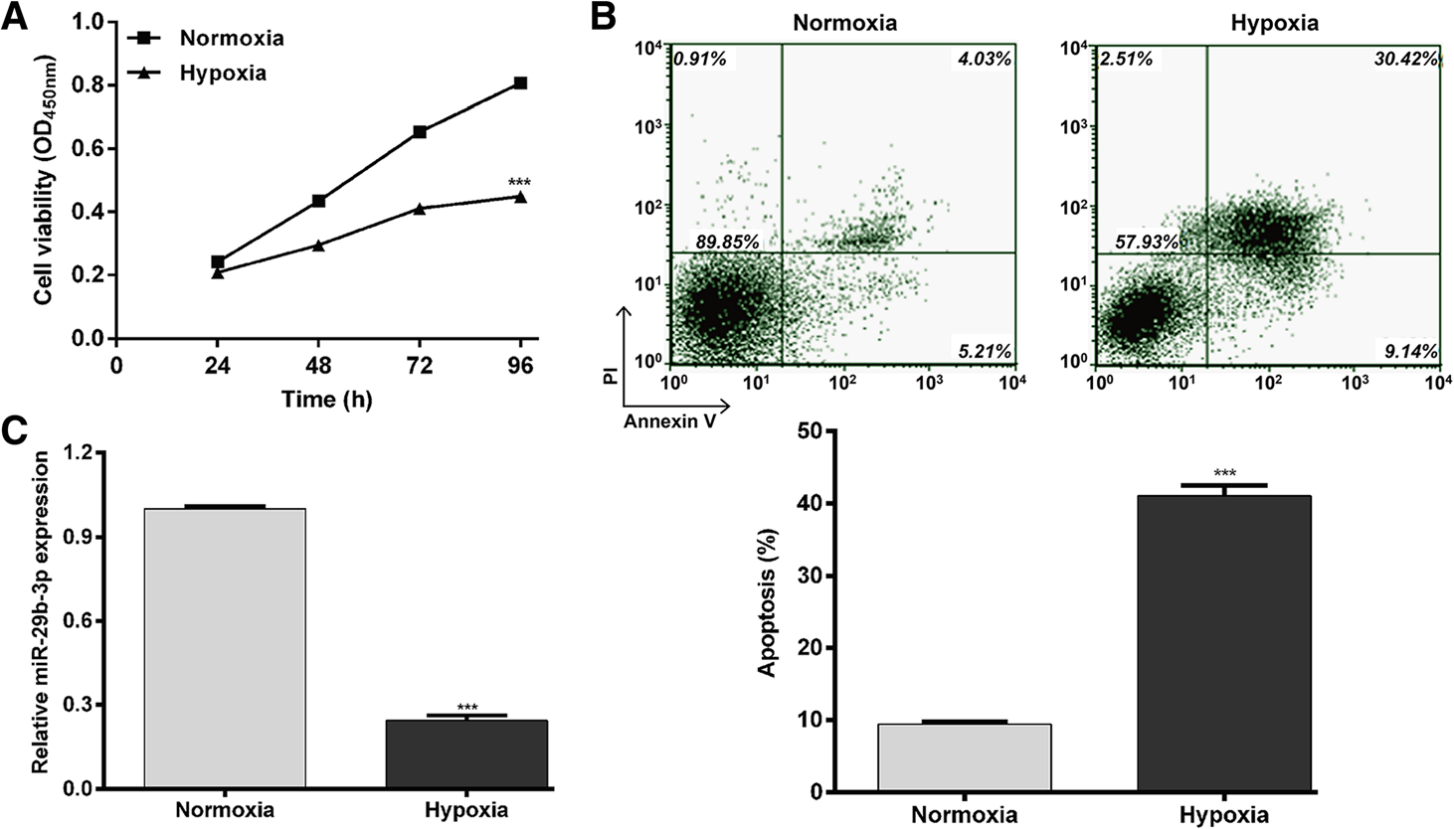
Hypoxia induces cell injury and downregulation of miR-29b-3p. AC16 cells were incubated in a hypoxic incubator containing 1% O_2_, 5% CO_2_ and 94% N_2_ to induce hypoxia. **a** – The cell viability of hypoxic and normoxic cells was measured using the CCK-8 assay. **b** – Apoptosis of hypoxic and normoxic cells was determined with a flow cytometry assay. **c** – The expression of miR-29b-3p was measured using quantitative real time PCR. Each experiment was repeated three times. ***p < 0.001 vs. normoxia

To investigate the potential function of miR-29b-3p in regulating hypoxic injury, we first induced hypoxia in vitro by incubating AC16 cells in 1% O_2_, 5% CO_2_ and 94% N_2_. Then, we analyzed the effects of hypoxia on cell viability and apoptosis in AC16 cells. Significant decreases in cell viability (*p* < 0.001 at 48, 72 and 96 h) were found post-hypoxia compared to the control normoxia group (Fig. 1a). Flow cytometry analysis further showed that the percentage of apoptotic cells was significantly higher in the hypoxia group than in the normoxia group (Fig. 1b; *p* < 0.001). Quantitative real time PCR analysis showed miR-29b-3p was markedly downregulated after hypoxia treatments compared to the normoxia group (Fig. 1c; *p* < 0.001). These findings indicate that downregulation of miR-29b-3p might play an important role in hypoxia-induced cell injury in AC16 cells.

### Upregulation of miR-29b-3p protected AC16 cells against hypoxia-induced cell injury

**Fig. 2:**
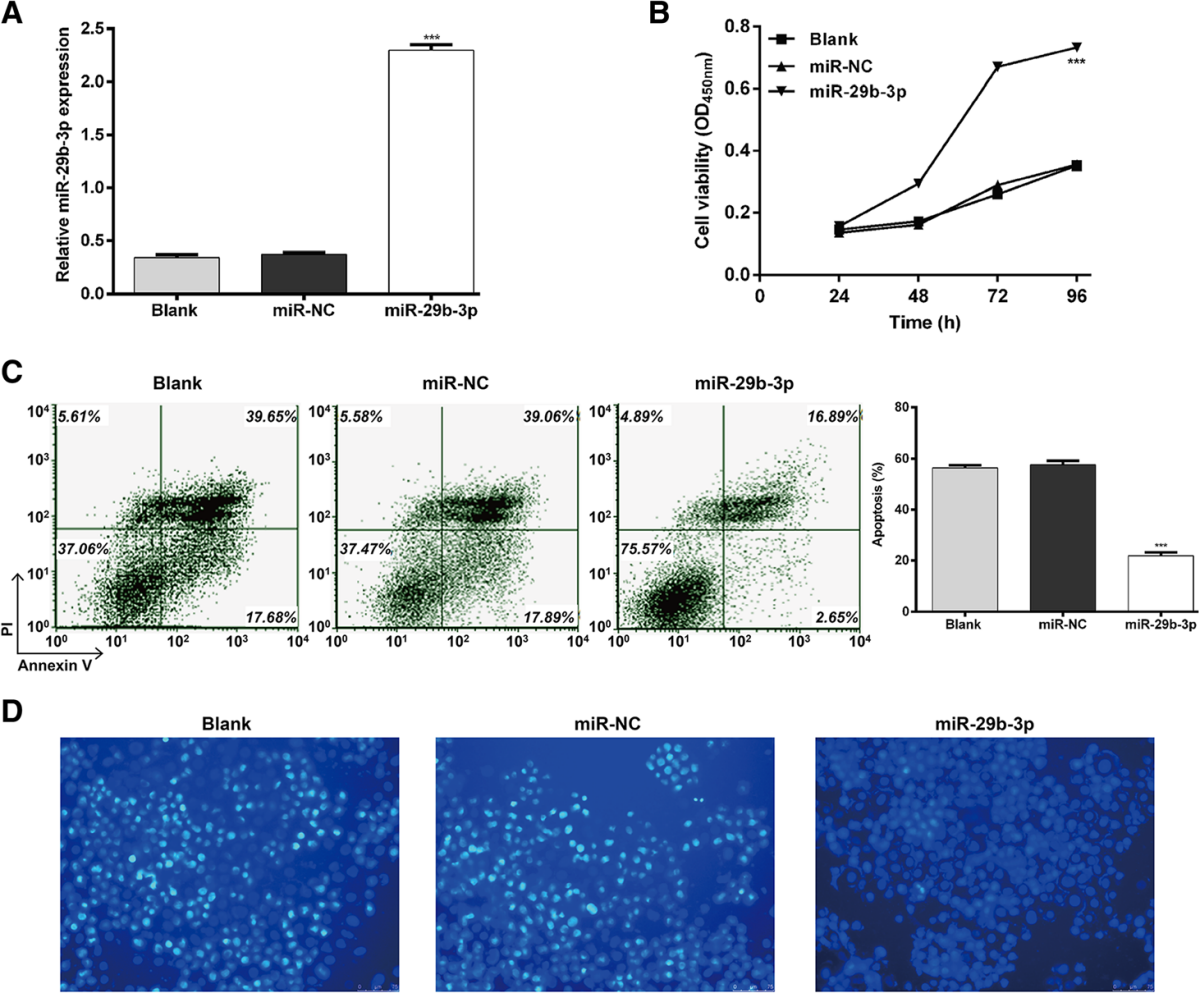
Hypoxia-induced AC16 cell injury is attenuated by miR-29b-3p overexpression. AC16 cells were transfected with miR-29b-3p or miR-NC before culture under hypoxic conditions. Non-transfected AC16 cells were cultured under normoxic conditions as a blank group. **a** – The expression of miR-29b-3p was detected using quantitative real time PCR. **b** – CCK-8 assay was performed to measure cell viability. **c** and **d** – Flow cytometry with Annexin V/PI staining and Hoechst 33258 staining were used to assess cell apoptosis. Each experiment was repeated three times. ***p < 0.001 vs. miR-NC or blank

MiR-29b-3p expression was markedly inhibited by hypoxia treatment, so we performed a gain-of-function assay to explore its biological function on hypoxic AC16 cells. The expression of miR-29b-3p was significantly elevated by transfection with miR-29b-3p compared to the miR-NC or blank control group (Fig. 2a; *p* < 0.001).

The CCK-8 assay showed miR-29b-3p overexpression could effectively reverse the impaired cell viability from hypoxic conditions (Fig. 2b; *p* < 0.001). Based on the results of Annexin V/PI staining (Fig. 2c; *p* < 0.001), the percentage of apoptotic cells decreased from 57.58% ± 2.80% in the miR-NC group to 21.83% ± 2.34% in the miR-29b-3p group. No significant difference was observed in the miR-NC and blank groups. Hoechst staining (Fig. 2d) was also employed for further evaluation of AC16 cell apoptosis. It showed consistent results with Annexin V/PI double staining. These results suggest that hypoxia-induced cell injury was attenuated by miR-29b-3p overexpression in AC16 cells.

### MiR-29b-3p downregulated TRAF5 expression by directly targeting its 3’UTR in AC16 cells

**Fig. 3:**
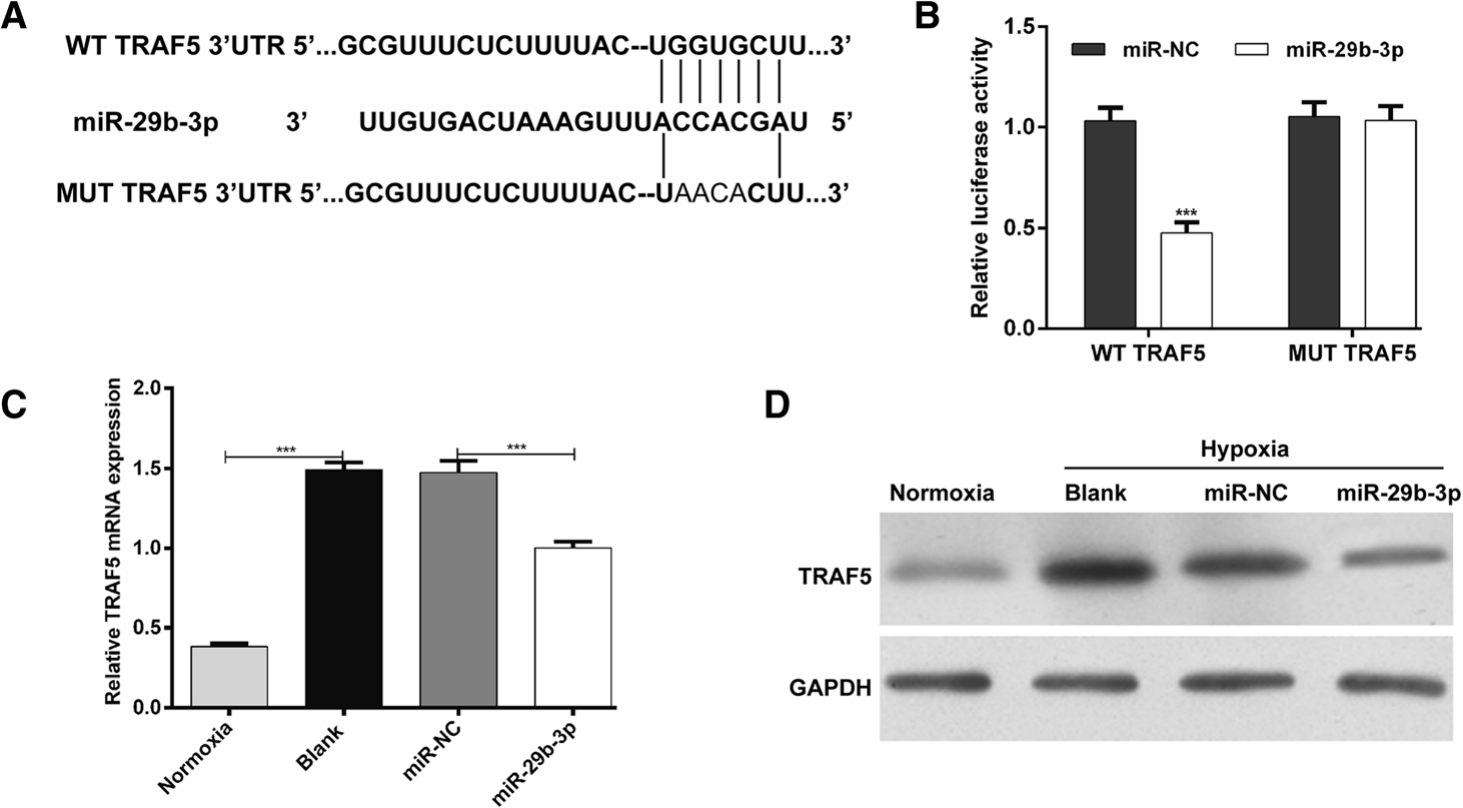
MiR-29b-3p targets the 3’UTR of TRAF5. **a** – The predicted miR-29b-3p-binding sites located in the 3’UTR of TRAF5. **b** – The effect of miR-29b-3p on luciferase activity was detected using the dual-luciferase reporter assay. The pmirGLO reporter vector containing the WT or MUT TRAF5 was co-transfected with miR-29b-3p or miR-NC into AC16 cells for 48 h. **c** and **d** – The mRNA and protein expressions of TRAF5 in AC16 cells transfected with miR-29b-3p or miR-NC and then subjected to hypoxia for 24 h were determined using quantitative real time PCR and western blot analysis. Each experiment was repeated three times. ***p < 0.001

To explore the underlying mechanism by which miR-29b-3p regulates hypoxic injury, we performed bioinformatics analysis to screen the potential target genes of miR-29b-3p. TNF receptor-associated factor 5 (TRAF5), an important regulator for cell survival [23], was predicted to bind to miR-29b-3p. The binding sequences are shown in Fig. 3a. To further test this, we sub-cloned the 3′-UTR of TRAF5 into the pmirGLO luciferase reporter. Transfection of miR-29b-3p significantly decreased the luciferase activity of the WT TRAF5 (Fig. 3b; *p* < 0.001), but did not obviously change the reporter activity of the pmirGLO luciferase reporter vector containing the MUT TRAF5. Furthermore, we detected the regulatory effects of miR-29b-3p on TRAF5 in AC16 cells. Both quantitative real time PCR (Fig. 3c, *p* < 0.001) and western blot (Fig. 3d) analysis showed that TRAF5 was significantly upregulated in hypoxia compared with normoxia. However, miR-29b-3p overexpression markedly suppressed the expression of TRAF5 in AC16 cells under hypoxia. Collectively, these results suggest that TRAF5 is a direct target gene of miR-29b-3p.

### Knockdown of TRAF5 potentiated the protective effects of miR-29b-3p against hypoxia-induced cell injury

**Fig. 4:**
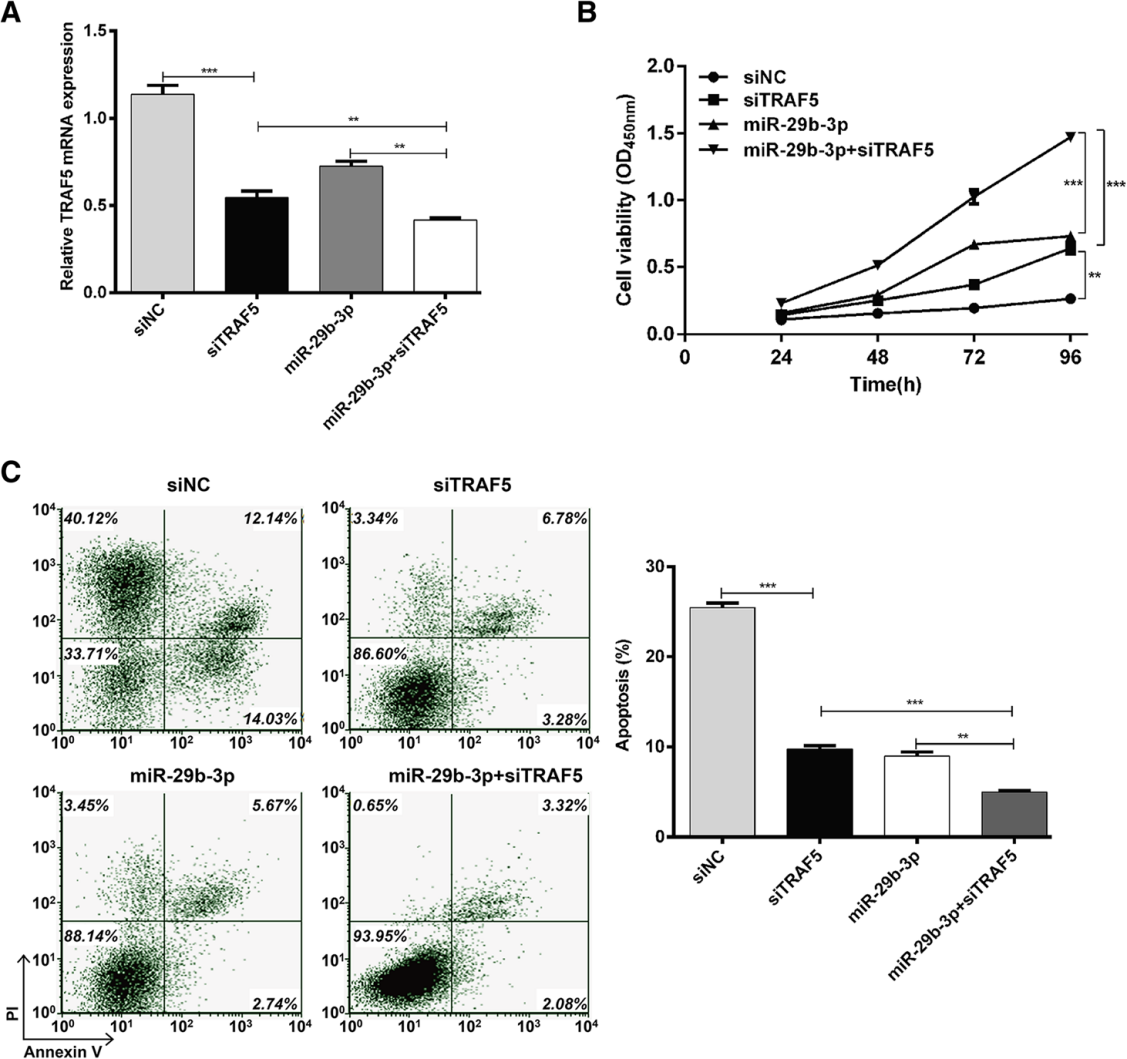
Knockdown of TRAF5 potentiated the protective effects of miR-29b-3p against hypoxia-induced cell injury. AC16 cells were transfected with siNC, siTRAF5 or miR-29b-3p, or co-transfected with miR-29b-3p and siTRAF5 before culture under hypoxic conditions. **a** – Expression of TRAF5 mRNA was detected using quantitative real time PCR. **b** – CCK-8 assay was performed to measure cell viability. **c** – Flow cytometry with Annexin V/PI staining was used to assess cell apoptosis. Each experiment was repeated three times. **p < 0.01, ***p < 0.001

Since TRAF5 was demonstrated to be upregulated in hypoxic conditions and suppressed by miR-29b-3p in AC16 cells, we investigated the biological function of TRAF5 to see if its knockdown could enhance the protective effects of miR-29b-3p against hypoxia-induced cell injury. AC16 cells were transfected with siNC, siTRAF5 or miR-29b-3p or co-transfected with miR-29b-3p and siTRAF5 before culture under hypoxic conditions. The expression of TRAF5 was confirmed to be significantly downregulated in AC16 cells after transfection with siTRAF5 or miR-29b-3p alone (Fig. 4a; *p* < 0.001). Notably, co-transfection with miR-29b-3p and siTRAF5 minimized the expression of TRAF5 (*p* < 0.01).

The CCK-8 assay (Fig. 4b) showed that the cell viability of AC16 cells increased after transfection with siTRAF5 or miR-29b-3p (*p* < 0.001), but a more significant increase was observed in co-transfected cells compared with two single transfections (*p* < 0.01).

Flow cytometry analysis was used to detect effects on cell apoptosis. TRAF5 knockdown significantly reduced the percentage of apoptotic cells (Fig. 4c; p < 0.01, p < 0.001), with a similar effect to miR-29b-3p overexpression (p < 0.001). Interestingly, we found that co-transfection with miR-29b-3p and siTRAF5 had more significant effects on cell apoptosis than two single transfections (p < 0.01, p < 0.001). These results further demonstrate that TRAF5 knockdown could strengthen the protective effects of miR-29b-3p against hypoxia-induced cell injury in AC16 cells.

## Discussion

Acute myocardial infarction (AMI) typically occurs as a consequence of myocardial necrosis caused by ischemia hypoxia. It affects more than 7 million people worldwide with a one-year mortality rate of approximately 10% [24]. Accumulating studies have showed that the regulation of target mRNAs by miRNAs plays an indispensable role in cardiac function [25].

MiR-29b-3p suppresses cell proliferation, invasion and epithelial–mesenchymal transition in bladder cancer [14]. A previous study revealed that miR-29b-3p could mechanistically directly bind to PGRN, accelerating chondrocyte apoptosis and contributing to the occurrence and progression of osteoarthritis [15]. Arterial calcification is characterized as an important pathological risk factor for cardiovascular disease, and it is associated with miR-29b-3p targeting matrix metalloproteinase-2 [26]. However, the further biological function of miR-29b-3p in AMI remains elusive.

In this study, we first confirmed that hypoxia induces injury in AC16 cardiomyocytes by decreasing cell viability and increasing apoptosis. We also found that hypoxic stress downregulated miR-29b-3p in cardiomyocytes. Moreover, miR-29b-3p was found to protect cardiomyocytes against hypoxia-induced cell injury.

Importantly, miRNAs are vital regulators of mRNA expression levels by binding to complementary sites within their target genes [27]. Using informatics-based prediction, we chose TRAF5 as a potential target gene for further investigation. Our loss- and gain-of-function analyses showed that suppression of TRAF5 potentiated the protective effects of miR-29b-3p against hypoxia-induced cardiomyocyte injury, suggesting a anti-proliferative and pro-apoptotic effects of TRAF5. Notably, we found miR-29b-3p induces additional protective effects on cardiomyocytes upon knockdown of TRAF5, suggesting that miR-29b-3p might inhibit apoptosis through regulation of other genes. In fact, TRAF5 is just one of the target genes of miR-29b-3p.

The TRAF family consists of 6 members: TRAF1 through TRAF6 [28]. Knockdown of TRAF5 was shown to reverse the suppression of miR-26b downregulation on the proliferation of esophageal squamous carcinoma cells [29]. In melanoma cells, TRAF5 overexpression could obviously counteract the anti-proliferative effects of tumor cells caused by elevated miR-26b expression [22]. Consistent with previous studies, we further showed TRAF5 inversely regulated by miR-29b-3p might be an anti-proliferative gene in cardiomyocytes.

TRAF5 is also a mediator of NF-kB activation, which plays an essential role in repression of TNF-α-induced cell death [30]. Previous studies implied that TRAF5 was also involved in the SAPK/JNK and MAPK signaling pathways associated with apoptosis [22, 31]. Interestingly, TRAF family members have been shown to co-express with p75NTR and differentially regulate the NF-kB response and cell apoptosis [32]. Interaction between p75NTR and TRAF2/TRAF6 led to NF-kB activation and promoted apoptosis, while p75NTR-TRAF6 co-expression had an inhibitory impact of P75NTR-mediated NF-kB activation [32]. In this study, TRAF5 has a pro-apoptotic role in cardiomyocytes, but the concrete signaling pathway is not clear yet. Further investigation is required.

## Conclusions

Our results revealed that miR-29b-3p is a hypoxia response miRNA. Enforced expression of miR-29b-3p protects against AC16 cardiomyocyte injury partially through the targeting of TRAF5. This study may deepen our understanding of the pathological mechanism underlying AMI occurrence.

## Abbreviations

AMI: Acute myocardial infarction
miRNA: microRNA
TNFR: Tumor necrosis factor receptor
TRAF: TNFR-associated factor
UTR: Untranslated region

## References

[1] Jaeger C, Wildi K, Twerenbold R, Reichlin T, Rubini Gimenez M, Neuhaus JD (2016). One-hour rule-in and rule-out of acute myocardial infarction using high-sensitivity cardiac troponin I. Am Heart J.

[2] McKeever RG, Vearrier D, Jacobs D, LaSala G, Okaneku J, Greenberg MI (2015). K2--not the spice of life; synthetic cannabinoids and ST elevation myocardial infarction: a case report. J Med Toxicol.

[3] Sharp TE, Schena GJ, Hobby AR, Starosta T, Berretta RM, Wallner M (2017). Cortical bone stem cell therapy preserves cardiac structure and function after myocardial infarction. Circ Res.

[4] Seropian IM, Toldo S, Van Tassell BW, Abbate A (2014). Anti-inflammatory strategies for ventricular remodeling following ST-segment elevation acute myocardial infarction. J Am Coll Cardiol.

[5] Ye L, Chang YH, Xiong Q, Zhang P, Zhang L, Somasundaram P (2014). Cardiac repair in a porcine model of acute myocardial infarction with human induced pluripotent stem cell-derived cardiovascular cells. Cell Stem Cell.

[6] Lin S, Gregory RI (2015). MicroRNA biogenesis pathways in cancer. Nat Rev Cancer.

[7] Wang GK, Zhu JQ, Zhang JT, Li Q, Li Y, He J (2010). Circulating microRNA: a novel potential biomarker for early diagnosis of acute myocardial infarction in humans. Eur Heart J.

[8] Melman Yonathan F, Shah R, Danielson K, Xiao J, Simonson B, Barth A (2015). Circulating MicroRNA-30d is associated with response to cardiac resynchronization therapy in heart failure and regulates cardiomyocyte apoptosis. Circulation..

[9] Fiedler J, Thum T (2013). MicroRNAs in myocardial infarction. Arterioscler Thromb Vasc Biol.

[10] Ai J, Zhang R, Li Y, Pu J, Lu Y, Jiao J (2010). Circulating microRNA-1 as a potential novel biomarker for acute myocardial infarction. Biochem Biophys Res Commun.

[11] Huang W, Tian SS, Hang PZ, Sun C, Guo J, Du ZM (2016). Combination of microRNA-21 and microRNA-146a attenuates cardiac dysfunction and apoptosis during acute myocardial infarction in mice. Mol Ther Nucleic Acids.

[12] Yang X, Qin Y, Shao S, Yu Y, Zhang C, Dong H (2016). MicroRNA-214 inhibits left ventricular remodeling in an acute myocardial infarction rat model by suppressing cellular apoptosis via the phosphatase and Tensin homolog (PTEN). Int Heart J.

[13] Wang BW, Wu GJ, Cheng WP, Shyu KG (2014). MicroRNA-208a increases myocardial fibrosis via endoglin in volume overloading heart. PLoS One.

[14] Lv M, Zhong Z, Huang M, Tian Q, Jiang R, Chen J (2017). lncRNA H19 regulates epithelial-mesenchymal transition and metastasis of bladder cancer by miR-29b-3p as competing endogenous RNA. Biochim Biophys Acta Mol Cell Res.

[15] Chen L, Li Q, Wang J, Jin S, Zheng H, Lin J (2017). MiR-29b-3p promotes chondrocyte apoptosis and facilitates the occurrence and development of osteoarthritis by targeting PGRN. J Cell Mol Med.

[16] Drummond CA, Fan X, Haller ST, Kennedy DJ, Liu J, Tian J (2018). Na/K-ATPase signaling mediates miR-29b-3p regulation and cardiac fibrosis formation in mice with chronic kidney disease. PLoS One.

[17] Yang KC, Ma X, Liu H, Murphy J, Barger PM, Mann DL (2015). Tumor necrosis factor receptor-associated factor 2 mediates mitochondrial autophagy. Circ Heart Fail.

[18] Xie P (2013). TRAF molecules in cell signaling and in human diseases. J Mol Signal.

[19] Evans S, Tzeng HP, Veis DJ, Matkovich S, Weinheimer C, Kovacs A, et al. TNF receptor-activated factor 2 mediates cardiac protection through noncanonical NF-kappaB signaling. JCI Insight. 2018;3(3). 10.1172/jci.insight.98278 PMC5821173 29415884

[20] Qi H, Xia FN, Xie LJ, Yu LJ, Chen QF, Zhuang XH (2017). TRAF family proteins regulate autophagy dynamics by modulating AUTOPHAGY PROTEIN6 stability in Arabidopsis. Plant Cell.

[21] Tada K, Okazaki T, Sakon S, Kobarai T, Kurosawa K, Yamaoka S (2001). Critical roles of TRAF2 and TRAF5 in tumor necrosis factor-induced NF-kappa B activation and protection from cell death. J Biol Chem.

[22] Li M, Long C, Yang G, Luo Y, Du H (2016). MiR-26b inhibits melanoma cell proliferation and enhances apoptosis by suppressing TRAF5-mediated MAPK activation. Biochem Biophys Res Commun.

[23] Hui G, Fang L, Li Y, Du J, Bei Z, Xiu W (2018). miR-873 inhibits colorectal cancer cell proliferation by targeting TRAF5 and TAB1. Oncol Rep.

[24] Piepoli MF, Corra U, Dendale P, Frederix I, Prescott E, Schmid JP (2017). Challenges in secondary prevention after acute myocardial infarction: a call for action. Eur Heart J Acute Cardiovasc Care.

[25] Aurora AB, Mahmoud AI, Luo X, Johnson BA, van Rooij E, Matsuzaki S (2012). MicroRNA-214 protects the mouse heart from ischemic injury by controlling ca(2)(+) overload and cell death. J Clin Invest.

[26] Jiang W, Zhang Z, Yang H, Lin Q, Han C, Qin X (2017). The involvement of miR-29b-3p in arterial calcification by targeting matrix Metalloproteinase-2. Biomed Res Int.

[27] Seok H, Ham J, Jang ES, Chi SW (2016). MicroRNA target recognition: insights from transcriptome-wide non-canonical interactions. Mol Cells.

[28] Miggin SM, O'Neill LA (2006). New insights into the regulation of TLR signaling. J Leukoc Biol.

[29] Chen Z, Zhao L, Zhao F, Yang G, Wang J (2016). MicroRNA-26b regulates cancer proliferation migration and cell cycle transition by suppressing TRAF5 in esophageal squamous cell carcinoma. Am J Transl Res.

[30] Beg AA, Baltimore D (1996). An essential role for NF-κB in preventing TNF-α-induced cell death. Science..

[31] Song HY, Regnier CH, Kirschning CJ, Goeddel DV, Rothe M (1997). Tumor necrosis factor (TNF)-mediated kinase cascades: bifurcation of nuclear factor-kappaB and c-Jun N-terminal kinase (JNK/SAPK) pathways at TNF receptor-associated factor 2. Proc Natl Acad Sci U S A.

[32] Ye X, Mehlen P, Rabizadeh S, VanArsdale T, Zhang H, Shin H (1999). TRAF family proteins interact with the common neurotrophin receptor and modulate apoptosis induction. J Biol Chem.

